# Supplementary material for: Studies on the Effects of Fermentation on the Phenolic Profile and Biological Activity of Three Cultivars of Kale
Source: Molecules. 2024 Apr 11;29(8):1727. doi: 10.3390/molecules29081727 (PMC11052505; doi:10.3390/molecules29081727)
Supplement: Supplementary file 1 [file molecules-29-01727-s001.zip › molecules-2903684-supplementary.pdf]

Table S1. Fingerprints of the analyzed extracts recorded in both negative (above) and positive (below) ion mode

Negative<br>-1<br>-2<br>-3<br>-4<br>-5<br>-6<br>-7<br>-8<br>-9	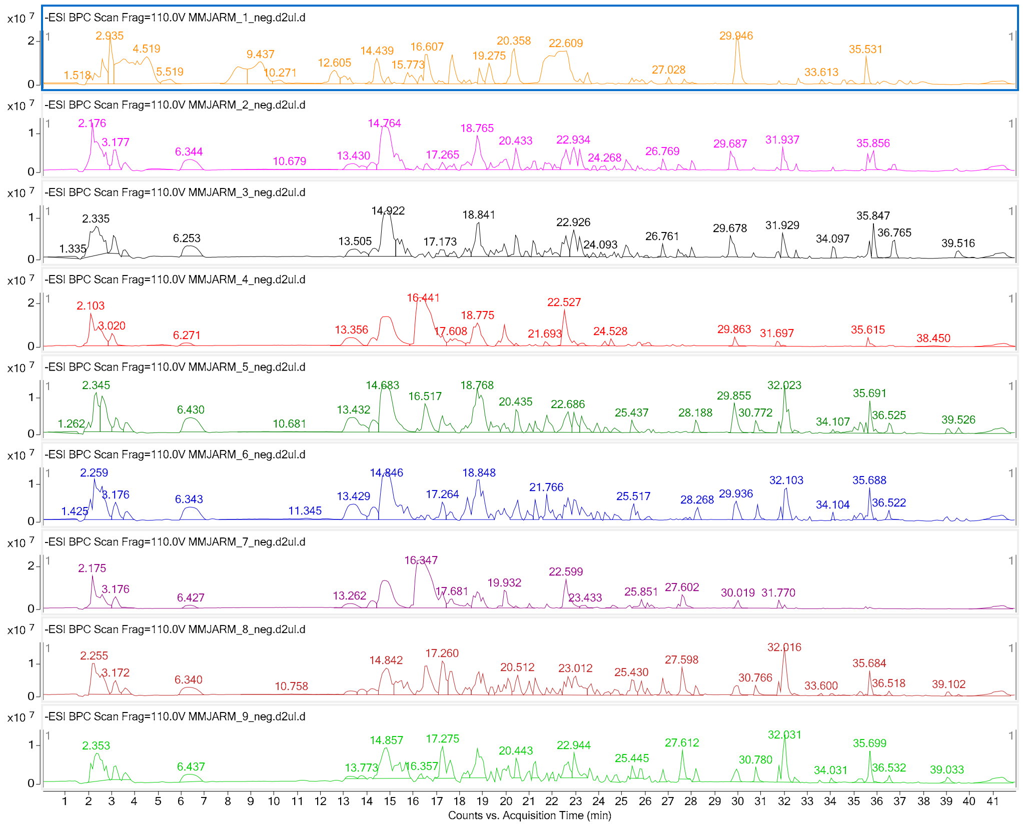
Positive<br>-1<br>-2<br>-3<br>-4<br>-5<br>-6<br>-7<br>-8<br>-9	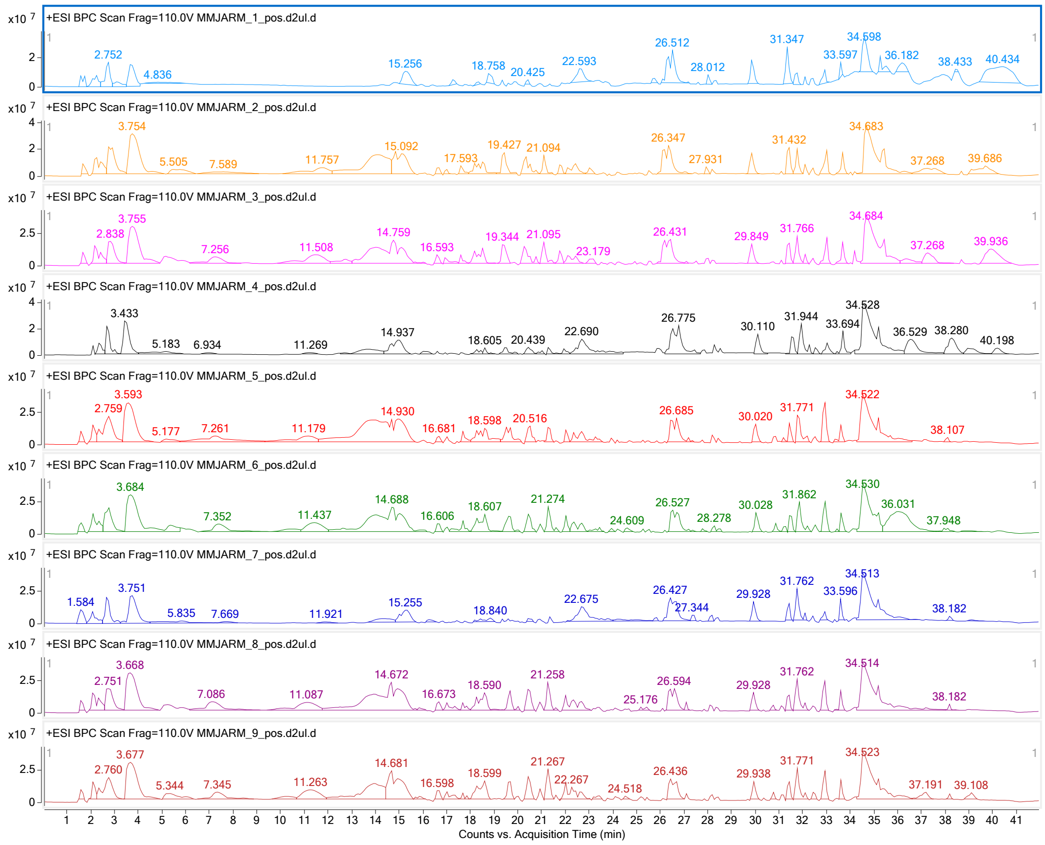
